# HIV and AIDS-related knowledge, HIV risk perception, uptake of HIV testing and its predictors among prison inmates in Bauchi State, Nigeria: a cross-sectional study

**DOI:** 10.11604/pamj.2023.46.7.38412

**Published:** 2023-09-07

**Authors:** Peter Okpeh Amede, Chukwuma David Umeokonkwo, Elizabeth Adedire, Muhammad Shakir Balogun, Sulaiman Saidu Bashir, Aisha Ahmed Abubakar

**Affiliations:** 1 Nigeria Field Epidemiology and Laboratory Training Program, Abuja, Nigeria,; 2 African Field Epidemiology Network, Abuja, Nigeria,; 3 Department of Community Medicine, Alex Ekweme University Teaching Hospital, Abakaliki, Ebonyi State, Nigeria,; 4 Department of Community Medicine, Ahmadu Bello University, Zaria, Nigeria

**Keywords:** HIV/AIDS, predictors, prisons, HIV testing, Nigeria

## Abstract

**Introduction:**

globally, HIV infection among prison inmates is significantly higher than in the general population. Therefore, it is important to identify inmates-living-with-HIV, through HIV-testing, in keeping with the target of UNAIDS vision 90-90-90. We assessed HIV/AIDS-related knowledge, HIV-risk perception and predictors of HIV-testing-uptake in Bauchi Prison.

**Methods:**

we conducted a cross-sectional study among 310 inmates selected with 2 stage sampling. Data was collected using a pretested, structured, interviewer-administered questionnaire. Inmates that consented to HIV-test were tested. We estimated inmates' knowledge about HIV/AIDS, the level of HIV testing uptake and predictors of HIV testing-uptake using multiple logistic regression at 5% significant level.

**Results:**

the mean age (SD) was 32.3 (±9.4) years and 94.8% were males, 47.1% (95% CI: 41.6-52.7) of the respondents had good knowledge of HIV/AIDS. Fifty-five percent (95% CI: 49.9-60.9) felt they were not at risk of contracting HIV. Uptake of HIV-testing was 58.1% (95% CI: 52.5-63.4). Independent predictors for the uptake of HIV-testing were age 35-44 years (aOR= 3.3; 95% CI: 1.4-7.7), positive risk perception (aOR= 3.3; 95% CI: 1.8-6.2), good knowledge of HIV (aOR= 9.6; CI: 5.1-18.0) and knowing someone who had died of AIDS (aOR= 4.1; 95% CI: 1.9-6.4).

**Conclusion:**

good knowledge of HIV/AIDS and HIV-testing-uptake was low among the inmates. We recommended the development and implementation of targeted HIV-testing interventions that cater to the specific needs of different age-groups within the prison population. The prison authority should develop prison-specific health education programme and awareness campaigns aimed at promoting accurate HIV-risk perception, improving their knowledge and help inmates make informed decisions that will prevent them from contracting HIV.

## Introduction

The rates of HIV infection among prison inmates worldwide are significantly higher than those in the general population with an average estimate of three to four times as many prison inmates being infected as members of the general population [1]. Prison is a high-risk environment for HIV transmission [2], with various, factors contributing to the vulnerability of inmates to HIV infection. These factors range from the weak criminal justice system, institutional and societal neglect, social stigma, lack of resources for maintenance of existing penal institutions, marginalization, inadequate healthcare, overcrowding, poor classification of prison inmates, poor inmates knowledge of HIV/AIDS, sharing of unsterile sharps and high-risk sex [3].

HIV testing is the gateway to HIV prevention, treatment, care and support. It provides opportunity for individuals or couples to learn about HIV and AIDS, make informed choice, know their status, help them accept and cope with the test outcome. It enhances behavioural change and help to reduce stigma and discrimination. HIV testing rates in Nigeria are low. In 2017, only 15.1% of people aged within 15-49 years had tested and knew their results [4]. Nigeria aims to reach the Joint United Nations Programme on HIV and AIDS (UNAIDS) target with 90% of People Living with HIV and AIDS (PLWHA) knowing their status by 2020 and 95% by 2030 [5]. The Nigeria national strategic framework recently set a target to achieve 100% availability of HIV testing services in key populations such as the prisons [4]. Testing acceptance rates among prison inmates may be particularly low where testing is done in the view of other prison inmates, with inadequate counselling services and confidentiality measures, and with inadequate follow-up care, treatment and support for those testing positive [6]. Other factors that could determine HIV testing uptake among prison inmates, could be fear of stigma and discrimination and sometimes the coercive setting in the prisons may prevent inmates from testing if it is available only on request or from disclosing risky behaviours such as People Who Inject Drugs (PWID), men who have sex with men (MSM) or unprotected heterosexual sex [7]. Utilization of HIV testing services in prisons has not been extensively studied in the African region, including Nigeria.

The high prevalence of HIV and the high risk of the infection among prison population necessitate the identification of inmates living with the risk of HIV infection, providing HIV testing and appropriate service for HIV treatment and prevention. This study is in keeping with the first policy target of UNAIDS vision 90-90-90. And inmates who test positive shall be linked to treatment, care and support thereby improving their lives, reducing AIDS-related mortality, reducing potential transmission in the prisons and lowering healthcare cost for the prison authority. Findings from this study promised to contribute to the limited information on HIV/AIDS in Bauchi prison.

The questions for the study include: what is the level of HIV and AIDS- related knowledge among inmates of Bauchi Prison? What are the perceptions of inmates in Bauchi Prison about risks of contracting HIV? And what is the level of uptake of HIV test and its predictors among inmates in Bauchi Prison? Therefore, in this study, we assessed inmates' HIV/AIDS-related knowledge, examined the perception of HIV risk, and determined the factors that predict HIV testing uptake among offenders incarcerated in Bauchi Prison in order to inform programmes that improve the inmates' knowledge and encourages them to actively seek for HIV testing and know their status.

## Methods

**Study design:** we conducted a cross-sectional study among inmates in Bauchi Prison.

**Setting:** this study was conducted in Bauchi Prison from 18^th^ to 29^th^ January 2021. Bauchi Prison is one of the five main prisons under the Bauchi State prisons command. Bauchi Prison has a maximum holding capacity of 500 inmates. The inmates' population in Bauchi Prison was 1002 with occupancy rate of 200.4% as at January 2021. The prison has one barbershop for all inmates with one clipper for haircuts and traditional barbers who use locally made shaving knives and razor blades for barbering and manicure. HIV testing is not offered routinely to the inmates on admission into the prison or as part of discharge policy, but HIV test is done on those who presented to the clinic with clinical features suggestive of HIV/AIDS. On the average 36 HIV tests are conducted annually with the highest in the last 5 years (2016-2020) being 55 HIV tests in 2017 and the least within same period, 11 in 2016. Inmates that test positive for HIV are referred to Abubakar Tafawa Balewa University Teaching Hospital, Bauchi for treatment and follow-up. During the five-year period (2016-2020), there were 15 AIDS-related deaths in Bauchi Prison. HIV counselling, condom distribution or HIV-risk reduction programmes are not offered in Bauchi Prison. Occasionally, health talks on HIV and AIDS are given by the medical staff, Non-Governmental Organizations (NGOs) and Faith Based Organizations (FBOs).

Data was collected using a pre-tested structured interviewer-administered questionnaire (Annex 1) adapted from the National Agency for the Control of AIDS (NACA) prison assessment questionnaire for inmates on vulnerability factors and response to HIV/AIDS in the Nigerian Prisons Service [8], and HIV Testing Services (HTS) kits. The questionnaire sought information on respondents' socio-demographic characteristics, knowledge about HIV/AIDS transmission and prevention, personal HIV risk perception, and factors associated with HIV testing uptake. The questionnaire has five sections (Section 1 with variables on socio-demographic characteristics of respondents; Section 2 on knowledge of HIV and AIDS; Section 3 on HIV risky behaviours; Section 4 on HIV risk perception and Section 5 on HIV testing uptake). The questionnaire was pre-tested in Ningi prison, where it was administered to 10% of the sample size (31 inmates: 28 males and 3 females). This helped to assess the flow of the questions and comprehension by the respondents.

Four research assistants for data collection were trained for a day by the principal researcher, on their roles, responsibilities and expectations during the study which ensured uniformity during data collection. The research assistants obtained a written informed consent (Annex 2) from each respondent prior to the interview [8]. Blood sample collections were done by finger prick from consenting respondents by two trained counsellors. The trained counsellors provided pre-test counselling before proceeding. Inmates that had the HIV test were provided on-site test results along with post-test counselling. HIV testing was conducted based on the national guideline for HIV counselling and testing using the serial HIV testing algorithm [9]. Inmates who tested positive for HIV were referred to the Bauchi prison health facility for care, treatment and support.

**Sample size estimation:** the sample of 310 inmates was determined using the Cochran formula for calculating single independent proportions [10], after correcting for finite population less than 10,000 and adjusting for 10% non-response rate. The parameters used in calculating the minimum sample size were standard normal deviate at 95% Confidence Interval (1.96), proportion of prison inmates who perceived themselves at risk for HIV infection (47%) in a previous study [11], and 5% margin of error.

### Participants

**Study population:** all inmates convicted or awaiting trial in Bauchi prison. Selection included all inmates incarcerated in Bauchi prison for at least three months prior to the study and excluded inmates with psychiatric illness, inmates who were critically ill and inmates that did not give consent to participate.

**Figure 1:**
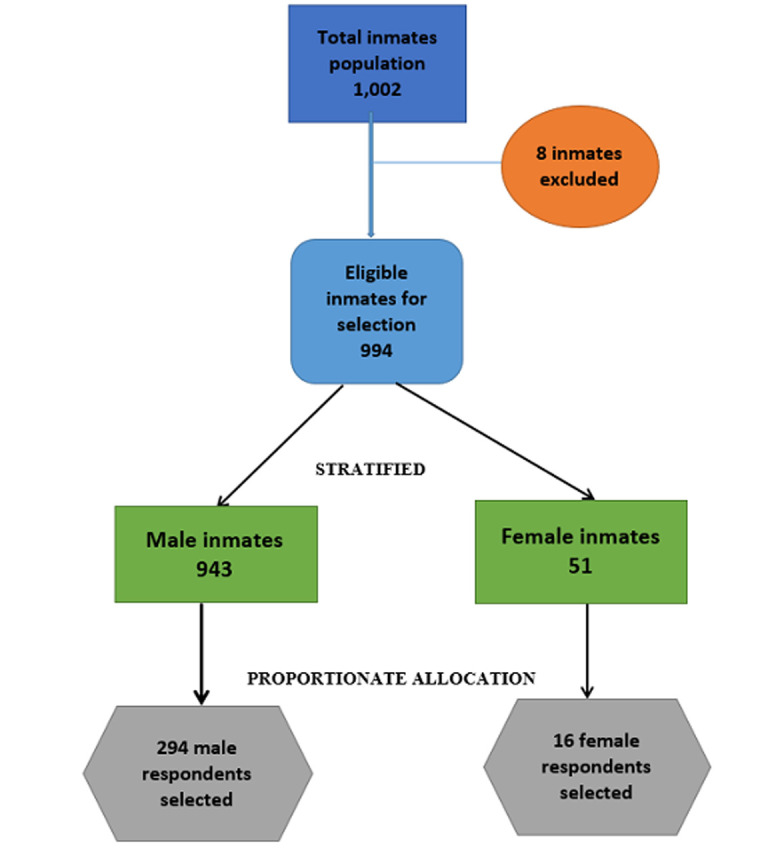
sampling flowchart

Two stage sampling technique was used to select the participants. In the first stage, Bauchi prison was randomly selected by balloting from the five main prisons (Bauchi, Azare, Ningi, Misau, and Jama'are) under Bauchi State prisons command and the second stage the study participants were then selected by stratified random sampling using computer-generated table of random numbers. The inmates were stratified into two groups; males and females. Proportionate allocation was employed to determine the number of participants needed from each stratum. Proportionate allocation ensures that the sample size from each of the stratum is representative of its proportion in the eligible population. Using the prison register, all eligible inmates were listed using the unique Prison Identification Number (PIN) assigned to each inmate upon first reception into the prison and allocated special numbers to form the sampling frame. There were 1,002 inmates, 8 were excluded (3 were critically ill and 5 were cases of active psychosis). Nine hundred and ninety-four were eligible (943 were males and 51 females) and all gave consent to participate. Using proportionate allocation; number of males divided by the total eligible participants multiplied by the sample size 943/994 X 310 = 294 male respondents and 16 (51/994 X 310) female respondents were randomly selected using computer generated random numbers. This method ensures that each inmate within the selected stratum has an equal chance of being included in the study (Figure 1).

Respondents were given the opportunity to seek clarification on any matter that was not clear to them. After the group address, individual interviews were conducted in secure rooms with some privacy within the prison to ensure the confidentiality of information provided by the respondents. Written informed consent was sought and obtained from respondents before the interview. The interviews were conducted either in the local language (Hausa) for those not fluent in English or English language for those that were fluent in the language. All study related biologic specimens and questionnaires were labeled with a study code. The respondent's identity (PIN and the individual study code) was captured on the survey questionnaires.

This study deployed the use of linked anonymous methods. Each sample was coded by the trained counsellors with the same code as on the respondent's questionnaire. All the respondents who consented were tested for HIV. They were also provided on site test results along with post-test Counselling with confidentiality. The trained counsellors provided pre-test counselling and obtained informed consent. The respondents were informed that the testing procedure would take 15-30 minutes. The trained counsellor performed serial HIV testing using Alere HIV-1/2 Determine^™^ as first line with a sensitivity of 100% and specificity of 99.68%. Individuals with positive test results were confirmed using HIV1/2 Stat-Pack (Chembio HIV 1/2 STAT-PAK^™^ Assay, Chembio diagnostic systems, Inc., Medford, NY, USA); which possesses a sensitivity/specificity of 99-100/100%. Unigold (Uni-GoldTM HIV, Trinity Biotech Plc, Co. Wicklow, Ireland) is used as a tiebreaker in cases of discordant tests, there were none recorded in this study.

**Variables:** the predictor variables are; sex, age, marital status, religion, educational status, knowledge of HIV and AIDS, knowing one who had died of AIDS, personal risk perception, duration of imprisonment and uptake of HIV test was the outcome variable. Good knowledge of HIV/AIDS was defined as awareness that having one faithful uninfected partner reduces the risk of HIV infection, that consistent use of condom during sexual intercourse prevents HIV infection, that HIV is not transmitted via mosquito bites, that healthy looking person can have HIV and, that HIV cannot be gotten through witchcraft or God's punishment. Risk perception is the subjective assessment of the individual's level of vulnerability to contracting HIV infection. Uptake of HIV test was defined as consenting and testing for HIV during the study period.

**Data sources/measurement:** the variables were measured directly with the questionnaire. However, some were categorized and redefined. Uptake of HIV test was measured on the nominal scale. The variable (uptake of HIV test) was dichotomized into Yes (respondents who consented and had the test) and No (those who didn't consent).

Knowledge about HIV and AIDS is a composite variable generated by the combination of five independent questions. knowledge about HIV and AIDS was assessed with five questions, knowing that consistent condom use during sexual intercourse and having just one faithful HIV-negative partner can reduce the chances of getting HIV, knowing that a healthy looking person can have HIV, and rejecting the two most common misconception about HIV transmission that HIV can be transmitted by mosquito bites and by supernatural means [12]. A right answer to any of the five questions was scored as one and a wrong answer scored zero. These were then summed up and scored over a total of five. A respondent who scored 5 was deemed to have good knowledge of HIV/AIDS and any score less than 5 was classified as not having a good knowledge of HIV/ AIDS [12].

Respondents were asked to assess their personal risk perception to contracting HIV infection. Those who said they perceived themselves to be at risk of contracting HIV were classified as at risk and those who said they were not at risk of contracting HIV were classified as not at risk or no risk. The variable age was measured directly using the questionnaire as single years as at respondents last birthday. These were then categorized as ten- year age-groups starting with 15-24, 25-34, 35-44 and 45 years or more. Duration of imprisonment was measured directly in months with the questionnaire and categorized into those who had been in prison less than 24 months and 24 months and above. Educational status was elicited directly and categorized into none; primary; secondary and tertiary.

**Data analysis:** data collected were coded and entered into a database. Data analysis was conducted using Epi Info version 7.2.5 (2021). Descriptive statistics were used to generate summary frequencies, percentages, means and standard deviation and presented in form of tables. Bivariate analysis was performed using Chi-square test to measure association between outcome variable (HIV testing uptake) and predictor variables (socio-demographic characteristics, Knowledge of HIV/AIDS, knowing one who had died of AIDS and HIV risk perception). The Chi-square test was used in this study as the outcome and predictor variables are both categorical. Covariates with p-value of ≤ 0.05 in the bivariate analysis were considered statistically significant and were included in the multiple logistic regression model to identify predictors of HIV testing uptake at 5% significant level while accounting for potential confounding variables.

**Ethical consideration:** ethical clearance was sought and obtained from Bauchi State Health Research Ethics Committee (BASHREC), with protocol approval number: NREC/03/11/19B/2020/42 before the commencement of the study. A written permission was sought and obtained from the Controller of Prisons, Bauchi State Command. Written informed consent was sought and obtained from the respondents before enrollment into the study and they were assured of confidentiality of their information. Respondents were informed that participation in both or either of the components (questionnaire and HIV testing) of the study is voluntary and they were reminded of their right to voluntary participation as well as their right to voluntary withdrawal even after initial consent. The refusal of participation by any eligible respondents did not attract any punishment or denial of benefit. All survey materials were not shared with those not connected with the study. Incentives given to respondents for participation were not encouraged and respondents who tested positive for HIV were linked to appropriate treatment and care. The information obtained was made anonymous and de-identified prior to analysis to ensure confidentiality.

## Results

**Socio-demographic characteristic:** a total of 310 respondents participated in the study with a response rate of 100% and all analyzed with no missing data. The mean age (SD) of respondents was 32.3 (±9.4) years, with a range from 18 to 60 years and the majority was male (94.8%). The median duration of imprisonment was 18 months (Inter quartile range: 10-28 months). About two-third, 204 (65.8%) had stayed in the prison for less than 2 years prior to the study (Table 1).

**Table 1:** socio-demographic characteristics of respondents in Bauchi Prison, 2021

Variable	Frequency (n = 310)	Percentage
**Age (years)**		
15-24	60	19.4
25-34	137	44.2
35-44	71	22.9
≥45	42	13.5
**Sex**		
Male	294	94.8
Female	16	5.2
**Religion**		
Muslim	246	79.4
Christian	64	20.6
**Education**		
None	101	32.6
Primary	40	12.9
Secondary	127	40.9
Tertiary	42	13.6
**Marital Status**		
Ever married	169	54.5
Never married	141	45.5
**Employment prior to imprisonment**		
Unemployed	71	22.9
Employed	239	77.1
**Employment type**		
Civil servants	30	12.6
Trading	67	28.0
Farming	67	28.0
Student	31	13.0
Artisan	35	14.6
Housewife	5	2.1
Female sex worker	4	1.7
**Duration of imprisonment**		
<24 months	204	65.8
≥24 months	106	34.2

**HIV and AIDS-related knowledge:** less than half 47.0% of the respondents had good knowledge of HIV and AIDS, majority are aware that consistent use of condoms during sexual intercourse (92.9%) prevent HIV transmission, that HIV cannot be gotten via witchcraft (79.9%) and 78.1% knew HIV cannot be transmitted by mosquitoes (Table 2). The odds of good knowledge of HIV among females is 0.3 times that of the males (OR=0.3; 95%CI: 0.1-0.8), age-group 35-44 years has a higher odds of good knowledge, inmates who attained tertiary education were 2 times more knowledgeable of HIV/AIDS compared to secondary educational attainment, those who attained secondary education has about 1.5 times odds of better knowledge compared to primary education and primary educational attainment is twice more knowledgeable compared to no education. The higher the educational level the more knowledgeable the individual is. The odds of good knowledge of HIV among inmates incarcerated for less than 2 years is 2 times that of inmates incarcerated for two or more years (OR=2.0; 95%CI: 1.2-3.3), but similar among the two religious groups and marital status (Table 3).

**Table 2:** HIV/AIDS-related knowledge of respondents in Bauchi Prison, 2021, n=310

Variable	Frequency (n=310)	Percentage
Awareness that having one faithful uninfected partner reduces risk of HIV infection	214	69.0
Awareness that consistent use of condom during sexual intercourse prevents HIV	288	92.9
Awareness that HIV is not transmitted via mosquito bites	242	78.1
Awareness that healthy looking person can have HIV	188	60.7
Awareness that HIV cannot be gotten through witchcraft or God’s punishment	247	79.9
Good knowledge		
Yes	146	47.1
No	164	52.9
**Multiple responses allowed**		

**Table 3:** relationship between knowledge of HIV/AIDS and respondents' socio-demographic characteristics

	Good knowledge		
	**Yes (%)**	**No (%)**	
**Variable**	**(n=146)**	**(n=164)**	**OR (95% CI)**
**Sex**			
Male	134 (45.6)	160 (54.4)	0.3 (0.1-0.8)
Female	12 (75.0)	4 (25.0)	1
**Age (years)**			
15-24	17 (28.3)	43 (71.7)	0.3 (0.1-0.7)
25-34	57 (41.6)	80 (58.4)	0.6 (0.3-1.2)
35-44	49 (69.0)	22 (31.0)	1.8 (0.8-4.0)
≥45	23 (54.8)	19 (45.2)	1
**Educational status**			
None	27 (27.0)	73 (73.0)	1
Primary	18 (43.9)	23 (56.1)	2.1 (1.0-4.5)
Secondary	72 (56.7)	55 (43.3)	3.5 (2.0-6.2)
Tertiary	29 (69.0)	13 (31.0)	6.0 (2.7-13.3)
**Marital status**			
Never married	64 (45.4)	77 (54.6)	1
Ever married	82 (48.5)	87 (51.5)	1.1 (0.7-1.8)
**Religion**			
Muslim	116 (47.2)	130 (52.8)	1.0 (0.6-1.8)
Christian	30 (46.9)	34 (53.1)	1
**Duration of imprisonment (years**)			
<2	108 (52.9)	96 (47.1)	2.0 (1.2-3.3)
≥2	38 (35.9)	68 (64.1)	1
**OR- Odds Ratio; CI- Confidence Interval**			

**HIV risk perception:** over half 55.5% of the respondents felt they were not at risk of contracting HIV infection. Only 44.9% of male and 37.5% of female respondents felt at risk to contracting HIV.

**Uptake of HIV test and its predictors:** measures of association between outcome and predictor variables both at bivariate and multivariate levels were performed. Factors associated with HIV testing uptake at bivariate level; age (p=0.03), marital status (p=0.01), risk perception (p<0.001), good knowledge (p<0.001), and knowing someone who died of AIDS (p<0.001) were statistically significant and so were modeled in multiple logistic regression (Table 4).

**Table 4:** association between respondents' socio-demographic factors and HIV testing uptake

	Uptake of HIV test			
	Yes(n=180)	No(n=130)		
Variable	n (%)	n (%)	P-value	OR (95% CI)
**Age (years)**				
15-24	44 (24.4)	16 (12.3)		3.3 (1.4-7.7)*
25-34	77 (42.8)	60 (46.1)		1.6 (0.8-3.1)
35-44	40 (22.2)	31 (23.9)		1.6 (0.7-3.4)
≥45	19 (10.6)	23 (17.7)	0.03*	1
**Sex**				
Male	171 (95.0)	123 (94.6)		1.1 (0.4-3.0)
Female	9 (5.0)	7 (5.4)	0.88	1
**Marital status**				
Ever married	87 (48.3)	82 (63.1)		0.5 (0.3-0.9)*
Never married	93 (51.7)	48 (36.9)	0.01*	1
**Educational status**				
None	56 (31.2)	44 (33.9)		0.8 (0.4-1.6)
Primary	26 (14.4)	15 (11.5)		1.1 (0.4-2.6)
Secondary	72 (40.0)	55 (42.3)		0.8 (0.4-1.6)
Tertiary	26 (14.4)	16 (12.3)	0.80	1
**Religion**				
Islam	149 (82.8)	97 (74.6)		1.8 (1.0-3.2)
Christian	31 (17.2)	33 (25.4)	0.08	1
**Duration of imprisonment**				
<2 years	122 (67.8)	82 (63.1)		1.2 (0.8-2.0)
≥2 years	58 (32.2)	48 (36.9)	0.39	1
**Risk perception**				
At risk	103 (57.2)	35 (26.9)		3.2 (2.0-5.3)*
No risk	77 (42.8)	85 (73.1)	<0.001*	1
**Good knowledge**				
Yes	119 (66.1)	27 (20.8)		7.4 (4.4-12.6)*
No	61 (33.9)	103 (79.2)	<0.001*	1
**Knowing someone who died of AIDS**				
Yes	140 (77.8)	67 (51.5)		3.3 (2.0-5.4)*
No	40 (22.2)	63 (48.5)	<0.001*	1

* Statistically significant; OR- Odds Ratio; CI- Confidence Interval

Predictors of uptake of HIV testing were; age, good knowledge, personal risk perception and knowing someone who died of AIDS after adjusting for other variables in the model to account for potential confounding effects. The results indicated that inmates who are 35-44 years were at least 3 times more likely to undergo HIV test (aOR= 3.3; 95% CI: 1.4-7.7) than inmates who are 45 years or older and twice more likely to test than inmates who are less than 35 years old (aOR=1.6; 95% CI: 0.7-3.1). Likewise, inmates who felt at risk of contracting HIV were at least 3 times more likely to undergo HIV test (aOR= 3.3; 95% CI: 1.8-6.2) than inmates who perceived themselves at no risk of contracting HIV. Similarly, inmates with good knowledge were about 10 times more likely to carry out HIV test (aOR= 9.6; CI: 5.1-18.0) compared to those with poor knowledge. Also, inmates who knew someone who died of AIDS were 4 times more likely to undergo HIV test (aOR= 4.1; 95% CI: 1.9-6.4) than inmates who didn't know anyone who died of AIDS (Table 5).

**Table 5:** predictors of uptake of HIV testing among respondents in Bauchi Prison, 2021

	Uptake of HIV test		
Variable	Yes	No	aOR (95% CI)
	**n (%)**	**n (%)**	
**Age (years)**			
15-24	44 (24.4)	16 (12.3)	1.6 (0.7-3.4)
25-34	77 (42.8)	60 (46.1)	1.6 (0.7-3.1)
35-44	40 (22.2)	31 (23.9)	3.3 (1.4-7.7)**
≥45	19 (10.6)	23 (17.7)	1
**Marital status**			
Ever married	87 (48.3)	82 (63.1)	0.8 (0.4-1.7)
Never married	93 (51.7)	48 (36.9)	1
**Risk perception**			
At risk	103 (57.2)	35 (26.9)	3.3 (1.8-6.2)**
No risk	77 (42.8)	85 (73.1)	1
**Good knowledge**			
Yes	119 (66.1)	27 (20.8)	9.6 (5.1-18.0)**
No	61 (33.9)	103 (79.2)	1
**Know someone who died of AIDS**			
Yes	140 (77.8)	67 (51.5)	4.1 (1.9-6.4)**
No	40 (22.2)	63 (48.5)	1

aOR- adjusted Odds Ratio; **Predictors for uptake of HIV test

## Discussion

The study assessed HIV and AIDS related-knowledge, HIV-risk perception, uptake of HIV testing and its predictors among inmates in Bauchi Prison. HIV testing is a crucial component of HIV prevention, treatment, and care. Timely diagnosis and linkage to care not only improve health outcomes for the inmates living with HIV but also play a significant role in preventing new infection. Understanding the influence of knowledge of HIV and HIV risk perception on HIV testing uptake is essential for developing effective HIV prevention strategies. By addressing knowledge gaps and promoting accurate risk perception, public health interventions can contribute to increasing testing rates.

The inmates' knowledge about HIV and AIDS was low, with low personal risk perception for HIV infection. HIV testing uptake was below the national and global target. The independent predictors of HIV testing uptake were; age, good knowledge, risk perception and knowing someone who died of AIDS. Knowledge, attitude and practice regarding HIV/AIDS are the corner stones in the fight against the disease. Good knowledge about HIV/AIDS is a powerful means of promoting positive attitudes and engaging in safe practices.

Good knowledge of HIV transmission, help individuals overcome misconceptions that could prevent behavioural change towards safe practices and reduce stigma against PLWHA. One-fifth of inmates in this study had some misconceptions about HIV/AIDS transmission such as the wrong beliefs that mosquito bites, supernatural means and the sharing of eating utensils are routes of transmission. These misconceptions, among others, have been documented among inmates in previous studies in different parts of the country [13-15]. A good number of inmates will eventually return to the community within few months or years. As such, health beliefs within the prison premises are strongly linked to health beliefs and practices in the wider society, necessitating the need to revisit the misconceptions surrounding HIV/AIDS existence and transmission among prison inmates.

In general, knowledge was low, hence, unsatisfactory among Bauchi Prison inmates. Worthy of note is that the female inmates demonstrated significantly higher level of good knowledge of HIV/AIDS than the males. The unsatisfactory knowledge reported in the study is a great threat to effective HIV/AIDS prevention and control programme in Bauchi Prison. As such, prisons-specific educational programmes should be well coordinated at regular intervals to facilitate more acceptances of interventions and to check inmates' practices that may undermine efforts in curbing the spread of HIV in this key population. In particular, and consistent with previous studies in Enugu [16] and Kaduna [17], older inmates with higher educational exposure and who had spent less than two years in prison, reported higher comprehensive knowledge of HIV/AIDS.

Less than half of the inmates believed that they were at risk of HIV infection. This finding is similar to that among youths in Cameroon [18], inmates in Ogbomoso prison [15], and that reported among inmates in the six geopolitical zones in Nigeria [11]. Not willing to access HIV test could result to people being diagnosed late, when the infection may have spread to others and progressed to AIDS when treatment is less effective. Above half of the prison inmates expressed their desire to be tested for HIV. This is lower than the level of uptake reported in Ogbomoso prison, but higher than that reported in a South-African correctional centre where only half would utilize the HIV Testing Services (HTS) [19]. HIV testing is crucial to the prevention of HIV/AIDS among high-risk groups. Consequentially, fear of stigmatization that are displayed towards PLWHA might have worsened the interest of people to go for testing and declare their status, eventually leading to under-reporting of the infection, increased spread, poor treatment access, and inadequate care and support programmes especially for this at-risk population. One of the objectives of the Revised Nigeria National HIV and AIDS Strategic Framework which is in line with the first target of UNAIDS vision 90-90-90 is to ensure that 90% of people living with HIV know their status by 2020 and 95% by 2030. In this study, just above a quarter of the respondents that tested positive to HIV are aware of their status before the study. This is far lower than the UNAIDS and the Revised Nigeria National HIV and AIDS Strategic Framework target of 90% of PLWHA should know their status at the end of 2020 [5,20]. This is lower than that reported among PLWHA in Nigeria who knew their HIV status in 2018 and globally in 2020 [5,21]. This low level of awareness of HIV status by the respondents is due to the poor HIV testing uptake prior to the study. Efforts should be made to enhance HIV testing among inmates in Bauchi Prison.

The study showed that age, good knowledge, risk perception, and history of knowing someone whose death was attributed to HIV/AIDS were significant predictors of uptake of HIV testing. In keeping with the findings documented in previous studies which showed significant association between HIV knowledge score, age and uptake of HIV testing [22,23], it was also observed that being within the age range of 35-44 years and having good knowledge are independent variables that strongly influenced the uptake of HIV testing among prison inmates in Bauchi. This was quite envisaged as Bauchi prison inmates in this age range demonstrated relatively higher knowledge score in comparison to the younger inmates.

In similar vein, inmates with relatively higher risk perception are three times more likely to test for HIV and seek for counselling. Also, inmates who knew an HIV-infected individual that eventually died of the illness are four times more likely to uptake HIV testing. Findings are in agreement with the earlier study on high-risk individuals in South Africa which reported that knowing or having lost a relative/friend who died from HIV, strongly contributed to higher risk perception, and subsequently, higher tendency for HIV testing practices [24,25]. This is unsurprising because people tend to learn overtime from the mistakes of others having witnessed how HIV-infected relatives/friends gradually decline in health quality. Subsequently, people acquire more knowledge about the disease and are pushed to observing necessary norms for prevention and treatment such as routine voluntary counselling and testing. Thus, engaging HIV-positive peer counsellors (who are actively undergoing anti-retroviral therapy) in prisons and other closed settings might be very helpful in scaling up HIV testing and counselling among the sampled high-risk individuals.

Poor knowledge of HIV, low perceived risk of HIV infection, and low uptake of HIV testing pose significant challenge to public health efforts in combating the HIV epidemic. Inmates who tested positive for HIV had previously been undiagnosed. This means that HIV positive inmates could be transmitting the AIDS virus in the prison, as well as accelerating community-based HIV infection if they are released without a proper transition programme. HIV positive inmates were identified early in the stage of the disease and linked to treatment, care, and support, preventing the infection from progressing to AIDS and thus improving their lives, reducing AIDS-related mortality, preventing further transmission of the virus in the prison, and lowering prison healthcare costs. By addressing these implications, HIV transmission rate in the prison will reduce with the resultant improvement in the overall health outcomes of the inmates. The findings from this study have been communicated to the prison authority.

**Limitations:** the study might have been impacted by social desirability and response bias, effort was made to ease respondents' fears and they were assured that the information they give shall be de-identified and made anonymous and shall not be used against them, and the study included only inmates in Bauchi Prison, therefore findings may not be generalizable.

## Conclusion

This study concludes that good knowledge of HIV and AIDS and uptake of HIV testing was suboptimal. HIV testing uptake was significantly influenced by age, good knowledge, personal risk perception, and knowing someone who died of AIDS. We recommended the development and implementation of targeted HIV testing interventions that cater to the specific needs of different age groups within the prison population by the prison health providers. The prison authority should develop prison-specific health education programme and awareness campaigns aimed at promoting accurate HIV risk perception, improving knowledge about HIV and help inmates make informed decisions that will prevent them from contracting HIV.

### What is known about this topic

- *HIV testing enable individual know their status and enhance behavioural change;*
- *Good knowledge of HIV/AIDS has proven to reduce HIV-related risk behaviours among individuals*.

### What this study adds

- *This study confirms the low rates of HIV testing uptake among inmates in Bauchi prison;*
- *Only 28.6% of HIV positive inmates in Bauchi prison knew their HIV status;*
- *Inmates who were incarcerated for less than 2 years were more knowledgeable than those imprisoned for 2 years and above*.

## Annexes

**Annex 1**: questionnaire on HIV and AIDS related-knowledge, risky behaviours, HIV testing uptake and its predictors among prison inmates in Bauchi State, Nigeria (PDF-782KB)

**Annex 2**: informed consent form (PDF-372 KB)

## References

[1] Adams LM, Kendall S, Smith A, Quigley E, Stuewig JB, Tangney JP (2013). HIV risk behaviors of male and female jail inmates prior to incarceration and one year post-release. AIDS and Behavior.

[2] Stone J, Fraser H, Lim AG, Walker JG, Ward Z, MacGregor L (2018). Incarceration history and risk of HIV and hepatitis C virus acquisition among people who inject drugs: a systematic review and meta-analysis. Lancet Infect Dis.

[3] United Nations Office on Drugs and Crime HIV and Prisons in sub-Saharan Africa: Opportunity for action.

[4] National Bureau of Statistics (NBS) and United Nations Children’s Fund (UNICEF) Multiple Indicator Cluster Survey 2016-17, Survey Findings Report.

[5] United Nations Office on Drugs and Crime HIV and AIDS - Nigeria.

[6] Hoxie NJ, Vergeront JM, Frisby HR, Pfister JR, Golubjatnikov R, Davis JP (1990). HIV seroprevalence and the acceptance of voluntary HIV testing among newly incarcerated male prison inmates in Wisconsin. Am J Public Health.

[7] Rosen DL, Schoenbach VJ, Wohl DA, White BL, Stewart PW, Golin CE (2009). Characteristics and behaviors associated with HIV infection among inmates in the North Carolina prison system. Am J Public Health.

[8] AIDS (NACA) NAFCOA (2009). Vulnerability factors and response to HIV and AIDS in the Nigerian Prisons Service: Zero draft.

[9] Federal Ministry of Health, Abuja Nigeria National AIDS and STIs Control Programme: National guidelines for HIV prevention, treatment and care.

[10] Abramson JH (2011). WINPEPI updated: computer programs for epidemiologists, and their teaching potential. Epidemiol Perspect Innov.

[11] UN Office on Drugs and Crime (2018). National Situation and Needs Assesstnent of HIV and AIDS, Drug Use and Related Health Services in Nigerian Prisons. National Agency for the Control Of AIDS.

[12] National Population Commission Abuja, Nigeria Nigeria Demographic and Health Survey 2018.

[13] Dolan K, Wirtz AL, Moazen B, Ndeffo-mbah M, Galvani A, Kinner SA (2016). Global burden of HIV, viral hepatitis, and tuberculosis in prisoners and detainees. Lancet.

[14] Audu O, Ogbooni SJ, Abdullahi AU, Sabitu K, Abah ER, Enokela OP (2012). Sexual Risk Behaviour and Knowledge of HIV/AIDS among Male Prison Inmates in Kaduna State. North Western Nigeria.

[15] Simooya O, Sanjobo N (2001). ‘In But Free´-an HIV/AIDS intervention in an African prison. Cult Heal Sex.

[16] Saliu A, Akintunde B (2014). Knowledge, Attitude, and Preventive Practices among Prison Inmates in Ogbomoso Prison at Oyo State, South West Nigeria. Int J Reprod Med.

[17] Okeke C, Uzochukwu B, Nawfal E, Abada E (2018). Assessment of the Awareness, Attitude, and Practice of Prisoners in Enugu to Acquired Immune Deficiency Syndrome (AIDS) and Voluntary Counselling and Testing (VCT). J Middle East North Africa Sci.

[18] Sabitu K, Iliyasu Z, Joshua IA (2009). An assessment of knowledge of HIV/AIDS and associated risky behavior among inmates of Kaduna convict prison: the implications for Prevention Programmes in Nigerian Prisons. Niger J Med.

[19] Tarkang E, Kweku M, Pencille L (2017). Knowledge and attitudes regarding HIV/AIDS among male prison inmates in an urban setting in the Southwest region of Cameroon. J Sci Res Stud.

[20] Motshabi LC, Pengpid S, Peltzer K (2011). HIV counselling and testing utilisation and attitudes of male inmates in a South African prison. SAHARA J.

[21] National Agency for Control of AIDS. Federal Ministry of Health N (2017). National Strategic Framework on HIV and AIDS: 2017-20 NACA.

[22] World Health Organization (WHO) (2012). Service Delivery Approaches to HIV Testing and Counselling (HTC): A Strategic HTC Programme Framework. WHO.

[23] Khawcharoenporn T, Chunloy K, Apisarnthanarak A (2016). Uptake of HIV testing and counseling, risk perception and linkage to HIV care among Thai university students. BMC Public Health.

[24] Asaolu IO, Gunn JK, Center KE, Koss MP, Iwelunmor JI, Ehiri JE (2016). Predictors of HIV Testing among Youth in Sub-Saharan Africa: A Cross-Sectional Study. PLoS One.

[25] Gebrekristos HT, Lurie MN, Mthethwa N, Karim QA (2005). Knowledge and acceptability of HAART among TB patients in Durban, South Africa. AIDS Care-Psychol Socio-Medical Asp AIDS/HIV.

